# AGE STEREOTYPE INTERNALIZATION ACROSS AGE AND CULTURES

**DOI:** 10.1093/geroni/igad104.0893

**Published:** 2023-12-21

**Authors:** Nicole Long Ki Fung, Helene Fung

**Affiliations:** The Chinese University of Hong Kong, Hong Kong, Hong Kong; The Chinese University of Hong Kong, Hong Kong, Hong Kong

## Abstract

The stereotype embodiment theory suggests that people internalize age stereotypes when they become self-relevant. This study explored the relationship between age and age stereotype internalization across cultures. We recruited adults aged 30 to 99 years from Hong Kong (N=524, Mage= 62.49), the United States (N=492, Mage= 57.90), Germany (N=790, Mage=63.38), Czechia (N=617, Mage=62.05), and Taiwan (N=660, Mage=60.78). Participants rated their self-perception of aging and attitude towards older adults. Across all the items, we calculated an intra-personal correlation score between self-perception of aging and attitude towards older adults. This index serves as a proxy of age stereotype internalization. Among all cultures, Hong Kong (r=0.57) and Taiwan (r=0.58) have the highest levels of internalization, followed by Czechia (r=0.47) and Germany (r=0.45). The United States (r=0.40) has the lowest level of internalization. The relationship between internalization and age varied across cultures. In Germany, there was a cubic relationship between internalization and age (p <.001). Internalization decreased with age till 52 years old, then it went up with age till 79 years old and dropped again. In Taiwan, we observed a quadratic relationship between internalization and age (p <.001). Internalization increased with age till the age of 64, then started decreasing. In Czechia, internalization increased with age linearly (p <.001). In Hong Kong and the United States, internalization was not associated with age. Our findings suggest that the internalization of age stereotypes into the self depends on cultures and age. More cross-cultural and longitudinal studies should be conducted to understand the age stereotype internalization process.

